# Discharge Regimes Transition and Characteristics Evolution of Nanosecond Pulsed Dielectric Barrier Discharge

**DOI:** 10.3390/nano9101381

**Published:** 2019-09-26

**Authors:** Li Zhang, Dezheng Yang, Sen Wang, Zixian Jia, Hao Yuan, Zilu Zhao, Wenchun Wang

**Affiliations:** 1 Key Lab of Materials Modification, Dalian University of Technology, Ministry of Education, Dalian 116024, China; zhangli2013@mail.dlut.edu.cn (L.Z.);; 2 Key Laboratory of Ecophysics, College of Sciences, Shihezi University, Shihezi 832003, China; 3 College of Electrical Engineering and Control Science, Nanjing Tech University, Nanjing 211800, China; 4 Laboratoire des Sciences des Procédés et des Matériaux CNRS., Institut Galilée, Université Paris 13, Sorbonne Paris Cité, 93430 Villetaneuse, France

**Keywords:** nanosecond pulse, temporal-spatial spectra, breakdown mechanism, reduced electric field, vibrational population

## Abstract

Discharge regime transition in a single pulse can present the breakdown mechanism of nanosecond pulsed dielectric barrier discharge. In this paper, regime transitions between streamer, diffuse, and surface discharges in nanosecond pulsed dielectric barrier discharge are studied experimentally using high resolution temporal–spatial spectra and instantaneous exposure images. After the triggering time of 2–10 ns, discharge was initiated with a stable initial streamer channel propagation. Then, transition of streamer-diffuse modes could be presented at the time of 10–34 ns, and a surface discharge can be formed sequentially on the dielectric plate. In order to analyze the possible reason for the varying discharge regimes in a single discharge pulse, the temporal–spatial distribution of vibrational population of molecular nitrogen N_2_ (C^3^Π_u_, v = 0,1,2) and reduced electric field were calculated by the temporal–spatial emission spectra. It is found that at the initial time, a distorted high reduced electric field was formed near the needle electrode, which excited the initial streamer. With the initial streamer propagating to the dielectric plate, the electric field was rebuilt, which drives the transition from streamer to diffuse, and also the propagation of surface discharge.

## 1. Introduction

As an effective method to optimize the ionization efficiency, nanosecond pulsed discharge (NPD) has become an emerging technology to generate non-thermal plasma [1]. For the sharply pulse rising time, the electrons can be accelerated effectively [2]. Therefore, high energy efficiency, excellent thermal stability, and good discharge plasma distribution can be reached in nanosecond pulsed dielectric barrier discharge (NPDBD) [3,4]. Moreover, some applications have been widely exhibited in volatile organic compounds (VOCs) removal [5], sewage treatment [6,7], polymer modification [8,9], aerospace [10], biomedical [11], etc. More recently, the nanoparticles, such as silver [12], carbon [13], and cobalt [14], have been synthetized by NPDBD. The investigation of the discharge mechanism and optimize the energy regulation could help to parameterize during the nanoparticle preparation. 

For this, the dynamic investigation of NPD was studied numerically and experimentally in the last two decades [15,16,17], and one of the most concerned issues is the discharge mechanism in rapid electric field. Several breakdown mechanisms were investigated in NPD, for instance, streamer, Townsend, and runaway electron modes [17,18,19,20,21,22,23,24,25,26]. Townsend breakdown often involves in a diffuse manner of the electrode gap [25]. In this mode, the secondary electrons play an important role in the breakdown cycle, which were mainly produced in the process of ions transporting to the cathode. After the production of secondary electrons, the electron avalanche continues or even grows until the discharge establishes. The streamer discharge is one of the most common mechanisms of gas breakdown at high *pd* (*p* is the pressure in in standard atmosphere, and *d* is the gap distance in meters) with overvoltage, which states that the electron avalanches initiate with the seed electrons created by photoionization, and follow the positively charged trail left by the primary avalanche [25]. According to different development degrees of the streamer, there are several regimes of streamer discharge, such as, positive corona, filament in dielectric barrier discharge, spark discharge, and plasma bullet [22,23,24,27]. Runaway electron mechanism is appropriate for the ultrafast pulse excitation with high overvoltage at atmospheric pressure, in which X-ray can also be detected [28,29].

Generally, the NPD with the rapid gas breakdown is not in a single discharge mode. Therefore, discharge regime transitions can take place in discharge process [30,31,32,33,34]. Lo et al. [30] studied the streamer-to-spark transition generated by an overvoltage nanosecond pulsed discharge under atmospheric pressure air. The initial discharge in their experiment was a streamer phase with high voltage and high current, followed by a spark phase with a low voltage and a decreasing current in several hundreds of nanoseconds, and at last in the streamer-to-spark transition, the discharge contracted toward the channel axis and evolved into a highly conducting thin column. Stepanyan et al. [31,32] studied the transitions of streamer to filamentary in nanosecond surface dielectric barrier discharge in air at pressures of 1–6 bar. The discharge developed as a set of streamers at atmospheric pressure, and filament could be observed at high pressures and high voltage amplitude. Both the time- and space-resolved optical emission spectra were measured in the transition of streamer-to-filament. Glow regimes also have been observed in NPD, which developed through an initial cathode-directed streamer and followed by a return wave of potential redistribution. Pai et al. [33] found that pin electrode is beneficial to generate the wave propagation, because the electric field at the tip would be significantly higher than that for a plane electrode, and result in greater neutralization of the streamer head space charge, causing a greater potential drop to transmitting a return wave. Townsend regime was also considered as a possible regime to generated diffuse discharge. However, Townsend breakdown cannot develop sufficiently in NPD [34], because it hardly accomplished in several tens of nanoseconds, as the external applied electric field evolves much faster than the time scale for ions to move across the gap.

The temporal–spatial resolved diagnosis of plasma characteristics, including plasma spectra and ICCD images, are very important to understand the dynamics processes and rapid breakdown mechanism in NPDBD [34,35]. By these diagnoses, the reduced electric field, the vibrational population of N_2_ (C^3^Π_u_), plasma dynamic evolution, can be studied to investigate plasma processes. Due to the short radiation lifetimes and high excitation rates of N_2_ (C^3^Π_u_) and ${N_{2}}^{+}(B^{2}\Sigma_{u}^{+})$, it is possible to calculate the reduced electric field *E*/*N* (*E* is electrical field, *N* is gas number density, and N = 2.68 × 10^19^ cm^−3^ at atmospheric air) by the ratio of intensities of N_2_ (C^3^Π_u_ → B^3^Π_g_) and ${N_{2}}^{+}(B^{2}\Sigma_{u}^{+})$ → X^2^∑_g_^+^) states in different vibrational radiation bands [36]. By the refined calculation of the excitation and quenching rates of the N_2_ (C^3^Π_u_, v = 0,1,2…), the temporal-spatial evolution of *E/N* can be represented by the temporal–spatial emission spectra of NPD [37]. In this paper, we concentrate our attention on the rapid breakdown mechanism and temporal–spatial evolution dynamic of NPDBD in air using needle-plate electrode. The ICCD image and the temporal–spatial resolved spectra are measured. The evolution dynamic process of the discharge and the temporal–spatial distributions of the emission intensities of N_2_ (C^3^Π_u_ → B^3^Π_g_) and $N_{2}{}^{+}(B^{2}\Sigma_{u}^{+}$ → X^2^Σ_g_^+^) bands are investigated. In addition, to understand the energy transition and breakdown mechanisms, the temporal–spatial distributions of vibrational population of N_2_ (C^3^Π_u_, v = 0,1,2) states and the *E/N* are calculated using the temporal–spatial emission spectra.

## 2. Experimental Setup

**Figure 1:**
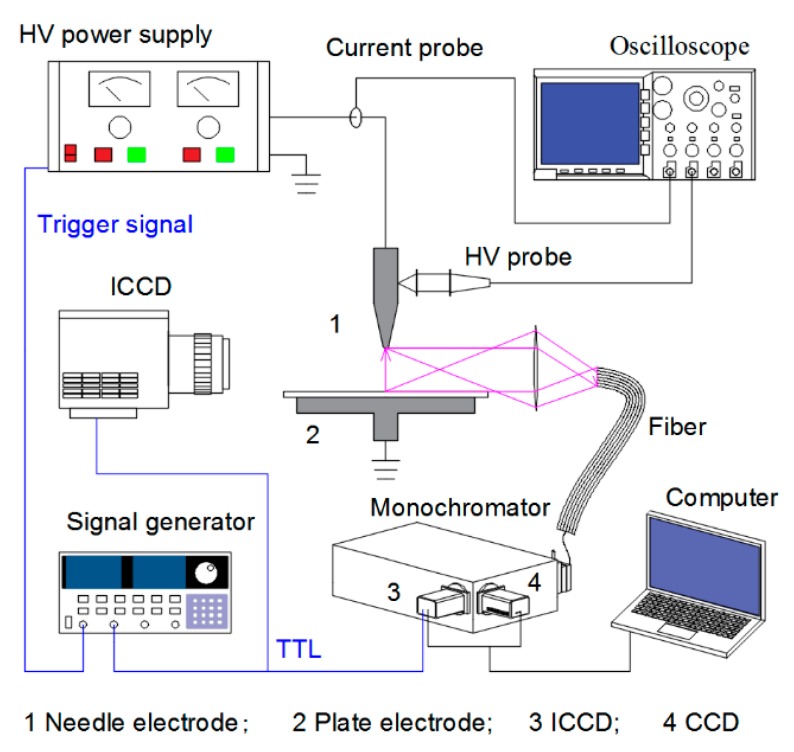
Experimental setup.

Figure 1 shows the schematic diagram of the experimental setup. The plasma reactor consists of a needle electrode with the curvature radius of 0.4 mm, and a grounded circular plate electrode covered by a 1 mm thick ceramic plate. The discharge is driven by a nanosecond pulsed power supply. Voltage probe (Tektronix- P6015A, Tektronix Inc, Beaverton, OR, USA) and current probe (Pearson Current Monitor-4100, Pearson Electronics Inc, Palo Alto, CA, USA) are used to measure the waveforms of pulse voltage and discharge current, which are displayed and recorded by an oscilloscope (Tektronix-TDS5054B-500 MHz, Tektronix Inc, Beaverton, OR, USA). The optical emission spectra are obtained using multichannel optical fibers and collected by a grating monochromator (Andor SR-750i, grating groove 2400 lines/mm, glancing wavelength 300 nm, Andor Technology Inc, Belfast, UK). A conjugate spectrum image system is used to acquire the spatially resolved optical spectra, where the heads of 35 parallel fibers are closely arranged in the vertical image plane of the quartz lens (*f* = 75 mm). After the diffraction of the grating, the output spectral light can be transformed into a digital signal by an intensified charge-coupled device (ICCD) camera (Andor’s iStar DH334T, Andor Technology Inc, Belfast, UK). For the time-resolved measurements, the ICCD camera is synchronized with the pulsed voltage.

## 3. Results and Discussion

### 3.1. Dynamics Evolution of the Discharge

**Figure 2:**
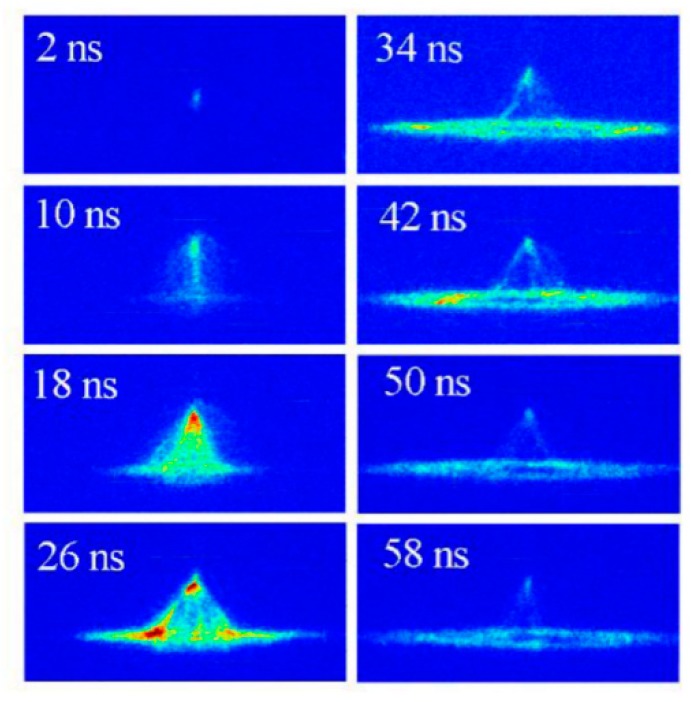
Images of discharge in air captured by an intensified charge-coupled device (ICCD) with an exposure time of 4 ns.

**Figure 3:**
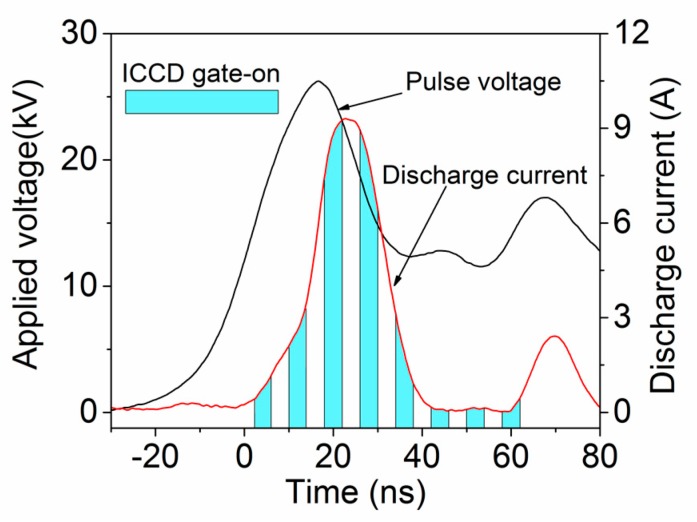
The waveforms of nanosecond pulsed voltage and discharge current.

A series of ICCD images with the gate width of 4 ns are shown in Figure 2, presenting the dynamics process of the gas breakdown in fast pulse electric field. The pulse peak voltage, pulse repetition rate, and electrode gap distance are kept at 26 kV, 100 Hz, and 5 mm, respectively. For a suitable synchronization, the pulse voltage and ICCD are triggered by a TTL signal. The time parameters labeled in Figure 2 are shown in the waveforms of pulse voltage and discharge current, illustrated in Figure 3, where the discharge current is obtained by subtracting the displacement current from the total discharge current [38,39]. The zero time in Figure 2 and Figure 3 is set at the initial breakdown of the gas gap. By applying a fast electric field higher than the breakdown field threshold, the local breakdown in the gap initiates from the needle electrode at 2 ns and exhibits a maximum intensity at the time of 18 ns. There are three main stages of the discharge development in NPDBD, i.e., streamer breakdown of the electrode gap, the streamer to diffuse transition, and the propagation of surface barrier discharge. Firstly, a main streamer, generated by the strong electric field, propagates from the needle to plate electrode at time t = 10 ns. It takes about 8 ns for the streamer channel to go across the gap, which means its velocity is about 6.25 × 10^5^ m/s. When the initial streamer propagated to the dielectric plate surface, the memory charges accumulated on the dielectric plate can be erased by the conductive streamer channel. Then, the electric field in the electrode gap and on the dielectric plate surface is rebuilt, and a secondary streamer channel can be generated and propagates along the dielectric plate. A number of fine secondary streamer channels distributing around the initial streamer channel can be observed from ICCD image at t = 10 ns. Numerous secondary streamer channels propagate both in horizontal and vertical directions in a synchronous manner, the overlap of these discharge channels makes the discharge presenting a diffusive morphology at the time of 10–18 ns. A non-uniform structure with several filaments can be observed at t = 26 ns. However, the pulse voltage is already decreasing and the volume discharge in gas gap becomes weak rapidly. Then, space charges are involved in the propagation of the streamer and in the enhancement of the induced field at the streamer head. Subsequently, this feedback electric field stops the ionization in the axial direction and builds a potential electric field gradient along the dielectric surface near the breakdown region. Therefore, the surface discharge can spread to the surrounding areas over 60 ns, when the volume discharge channel has been extinguished, that is, the discharge current in Figure 3 is approximate equal to zero.

The breakdown of the initial stage can be considered as a pulsed corona discharge in a non-uniform electric field. When the corona-like discharge is initialed at t = 2 ns, the electric field near the needle electrode can be estimated as *E_max_* = 2*V*/[*r*ln(2*d/r*)] ≈ 150 kV/cm [25], where *r* is the radius of the needle electrode, *d* is the gap distance along the axis, and *V* is the voltage at 2 ns. That means, *E/N* contributed by the applied pulse voltage is about 600 Td. This high electric field is strong enough to drive the gas gap breakdown as a positive streamer mode [33]. Once the initial streamer channel propagates across the gas gap and the streamer head reaches the cathode electrode, it can erase the memory charges on the dielectric plate surface where the discharge channel touched. During the breakdown in several nanoseconds, this time is not enough for the streamer–cathode interaction to fully transform into a cathode fall [33]. In other words, the non-metal cathode and short time are not sufficient to meet the condition of secondary electron emission. Thereby, the conductive plasma channel can be considered as an anode in which the *E/N* would become much smaller. Then, since the applied voltage is still in a high level, a newly built electric field with radial direction is formed to drive the subsequent breakdown. Meanwhile, the surrounding air can be pre-photoionized by the streamer channel. Although photoionization is orders of magnitude lower than the ionization density, it plays an important role in the propagation of the initial streamers [33,40,41,42]. Streamers can propagate nearly perpendicularly to the background electric field, and it can be guided by pre-ionization [40]. In addition, in the study of Nijdam [42] it is found that when the streamers do not follow the background field lines, they are usually repelled by neighboring streamers and follow the new local electric field direction. As a consequence, abundant of fine secondary streamer channels distributing around the initial streamer can be formed by pre-photoionization (as shown in Figure 2 at t = 10 ns), which contribute the transition to a diffuse regime.

### 3.2. Optical Emission Spectra of Nanosecond Pulsed Dielectric Barrier Discharge (NPDBD)

**Figure 4:**
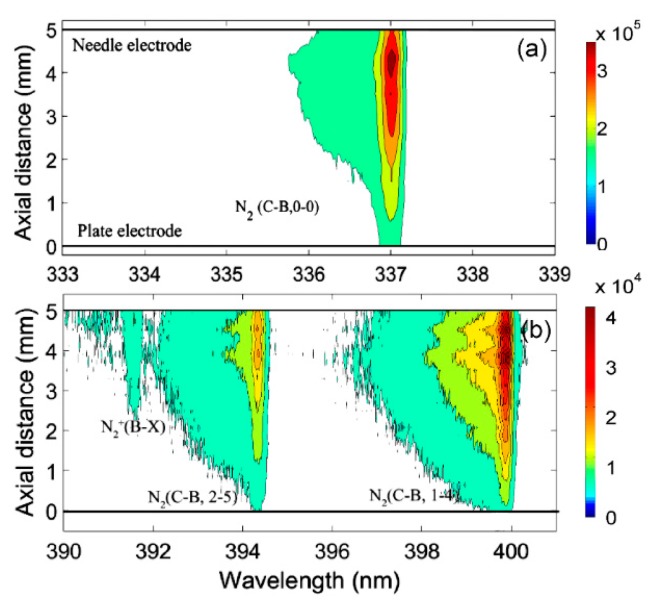
Spatial resolved spectra of nanosecond pulsed dielectric barrier discharge (NPDBD) (**a**) in range of 333–339 nm; (**b**) in range of 389–401 nm.

Figure 4a,b shows the optical emission spectra (OES) of NPDBD in the ranges of 333–339 nm and 390–401 nm at atmospheric air as a function of the distance on needle-plate axial direction. For the measurements in the experiment, the pulse peak voltage, pulse repetition rate, and electrode gap distance were kept at 26 kV, 100 Hz, and 5 mm, respectively. It can be seen in Figure 4 that both the emission intensities of $N_{2}{}^{+}(B^{2}\Sigma_{u}^{+}$ → X^2^Σ_g_^+^, 0–0) and N_2_ (C^3^Π_u_ → B^3^Π_g_, 0–0) exhibit the maximum value at the position about 1 mm from the needle tip and decrease as the distance from the needle tip increases. A significant difference between their spatial distribution is the spectra of $N_{2}{}^{+}(B^{2}\Sigma_{u}^{+}$ → X^2^Σ_g_^+^) mainly emitted from the region near the needle tip, which is much smaller than that of N_2_ (C^3^Π_u_ → B^3^Π_g_).

**Figure 5:**
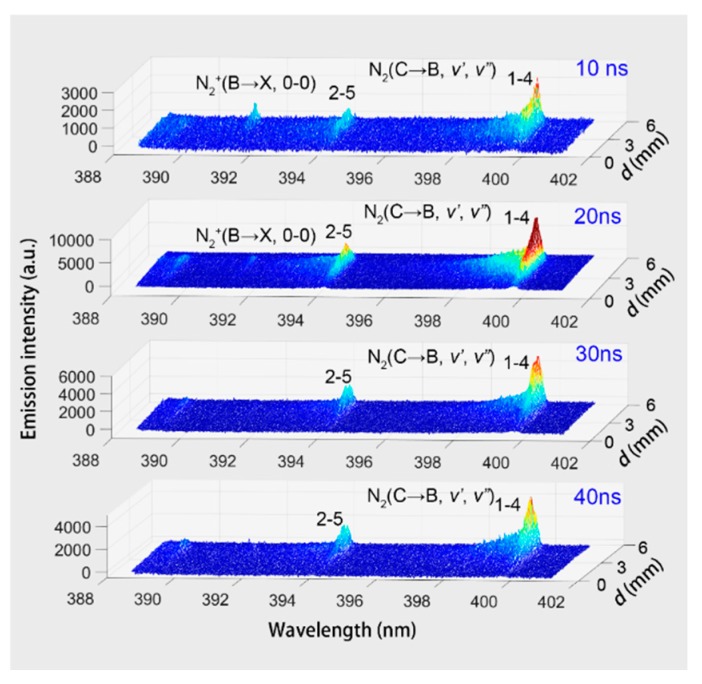
Spatial resolved spectra in the range of 389–401 nm at four different times.

According to the spatially resolved OES shown in Figure 4, spatial–temporal resolved spectra can be obtained by an ICCD detector. Figure 5 shows the transient spatial resolved OES in the range of 389–401 nm at four different times. During the measurement, the gate width of ICCD was kept at 5 ns, and the pulse peak voltage, pulse repetition rate, and electrode gap distance are kept at 26 kV, 100 Hz, and 5 mm, respectively. It shows that there is an obvious difference between the distribution of emission intensities of $N_{2}{}^{+}(B^{2}\Sigma_{u}^{+}$ → X^2^Σ_g_^+^, 0–0) and N_2_ (C^3^Π_u_ → B^3^Π_g_). When the discharge initiates from the needle electrode, the emission intensity of $N_{2}{}^{+}(B^{2}\Sigma_{u}^{+}$ → X^2^Σ_g_^+^, 0–0), which stands for the high reduced electric field, is approximated equal to the intensity of N_2_ (C^3^Π_u_ → B^3^Π_g_, 2–5). At the time of 30–40 ns, the bands of $N_{2}{}^{+}(B^{2}\Sigma_{u}^{+}$ → X^2^Σ_g_^+^, 0–0) become very weak.

**Figure 6:**
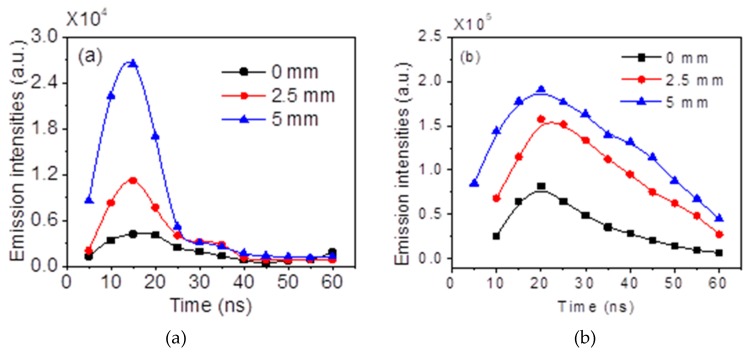
Spatiotemporal distribution of the emission intensities: (**a**) $N_{2}{}^{+}(B^{2}\Sigma_{u}^{+}$ → X^2^Σ_g_^+^, 0–0, 391.4 nm); (**b**) N_2_ (C^3^Π_u_ → B^3^Π_g_, 2–5, 394.3 nm).

Figure 6a,b shows the emission intensities of $N_{2}{}^{+}(B^{2}\Sigma_{u}^{+}$ → X^2^Σ_g_^+^, 0–0) and N_2_ (C^3^Π_u_ → B^3^Π_g_, 2–5) at 0 mm, 2.5 mm, and 5 mm from the plate electrode as functions of time in one single pulse, by integrating the spatial–temporal resolved spectra. The NPDBD was operated at atmospheric air at 26 kV pulse peak voltage, 100 Hz pulse repetition rate, and 5 mm electrode gap distance. The gate width of ICCD was set as 5 ns and the zero time is set at the initial breakdown of the gas gap. 

It can be seen from Figure 6a that once the breakdown initiated, the spectra of $N_{2}{}^{+}(B^{2}\Sigma_{u}^{+}$ → X^2^Σ_g_^+^, 0–0) is firstly emitted from the region near the needle electrode. Its emission intensity increases sharply at first 15 ns and then decrease, exhibiting a maximum at the time of 15 ns. The existence time of N_2_^+^ (B^2^Σ_u_^+^ → X^2^Σ_g_^+^, 0–0) is about 20 ns, which is only about 1/3 of the discharge duration time in a single pulse (in a characteristic time of 50–60 ns). Distance from the plate electrode has an obvious influence on the emission intensity of $N_{2}{}^{+}(B^{2}\Sigma_{u}^{+}$ → X^2^Σ_g_^+^, 0–0). When the distance from the plate electrode decreases from 5 mm to 2.5 mm, which means the detection region moves to the middle of the electrode gap from the region near the needle electrode, the emission intensity decreases sharply to about 1.2 × 10^4^ (a.u.). There is a little distinction in the evolution of $N_{2}{}^{+}(B^{2}\Sigma_{u}^{+}$ → X^2^Σ_g_^+^, 0–0) near the plate electrode. When the volume discharge is extinguished at 55–60 ns, a slight increase of the emission intensity of $N_{2}{}^{+}(B^{2}\Sigma_{u}^{+}$ → X^2^Σ_g_^+^, 0–0) can be observed (from 2156 to 9182).

As shown in Figure 6b, the temporal evolutions of N_2_ (C^3^Π_u_ → B^3^Π_g_, 2–5) spectral bands present an obvious different tendencies both on spatial and temporal dimensionality. Firstly, the time period for the emission of N_2_ (C^3^Π_u_ → B^3^Π_g_, 2–5) is much longer, it can almost be detected during the whole time from 10–60 ns. The maximum values of emission intensity of N_2_ (C^3^Π_u_ → B^3^Π_g_, 2–5) appears at the time of 20 ns, which slightly lag behind the $N_{2}{}^{+}(B^{2}\Sigma_{u}^{+}$ → X^2^Σ_g_^+^, 0–0) band. Secondly, the attenuation gradients of N_2_ (C^3^Π_u_ → B^3^Π_g_, 2–5) is much smaller. The emission intensity of N_2_ (C^3^Π_u_ → B^3^Π_g_, 2–5) at 5 mm from the plate electrode is about 1.2 times of the emission intensity at 2.5 mm, while the emission intensity of the $N_{2}{}^{+}(B^{2}\Sigma_{u}^{+}$ → X^2^Σ_g_^+^, 0–0) band at 5 mm is about 2.2 times of the emission intensity at 2.5 mm.

As observed in Figure 2, the discharge initialed from the needle electrode as a streamer mode. When the positive streamer propagates to the plate electrode, the streamer head with a distorted high electrical field can produce high energetic electrons with the main energy of about 2–20 eV [34], which can ionize and excite the N_2_ molecules to excited ions $N_{2}{}^{+}(B^{2}\Sigma_{u}^{+}$). The relative strong bands of $N_{2}{}^{+}(B^{2}\Sigma_{u}^{+}$ → X^2^Σ_g_^+^) can be emitted from the region near the needle electrode at the time of 10−20 ns. When the initial positive streamer develops sufficiently, the main streamer changes to diffuse mode. At this time, the discharge intensity arrives to the maximum (25 ns). However, since the local strong electrical field caused by the streamer breakdown would not exist, the *E/N* decrease to a low level, the electron energy is insufficient to ionize the N_2_ molecules, and the emission intensity of $N_{2}{}^{+}(B^{2}\Sigma_{u}^{+}$ → X^2^Σ_g_^+^,0–0) drops very low after t = 25 ns.

### 3.3. Time and Space Distribution of Vibrational Population of N_2_ (C^3^Π_u_, v = 0,1,2)

In atmospheric pressure air plasma, some energy can be transfused and stored in the molecule vibrational energy levels by the electron impact vibrational excitation process. Since the energy level gap of the molecules vibrations is in the scale of one-tenth eV (for N_2_ (C^3^Π_u_, v = 0) to N_2_ (C^3^Π_u_, v = 1) is about 0.22 eV), which is in the intermediate state between electronic energy (in the scale of several eV) and rotation energy, the vibrational energies of N_2_, O_2_, etc., play an important role in plasma energy transfer and plasma chemical processes [43].

For the specified vibrational transition from upper level v’ to the lower level v”, the emission intensity *I*_v’v”_ is proportional to the density of photon *n_f_*, which can be obtained by following Equations [27]:

*I*_v’v”_ ∝ d*n_f_*/ d*t* = N _v’_*(FC)*_v’v”_*(R_e_)*_v’v”_*v*_v’v”_ (1)

*I_0–3_*/*I_1–4_* = *N_0_(FC)_0–3_* (*Re*)*_0–3_v_0–3_*/*N_1_* (*FC*)*_1–4_* (*Re*)*_1–4_v_1–4_* (2)

Since *(Re)_v’v”_* is almost constant, the relative populations of N_2_ (C^3^Π_u_, *v* = 1) can be expressed as:

*N_1_*/*N_0_* = *I_1–4_*(*FC*)*_1–4_v_1–4_*/*I_0–3_*(*FC*)*_0–3_v_0–3_* (3)

The relative populations of N_2_ (C^3^Π_u_, v = 2) can also be calculated similarly.

**Figure 7:**
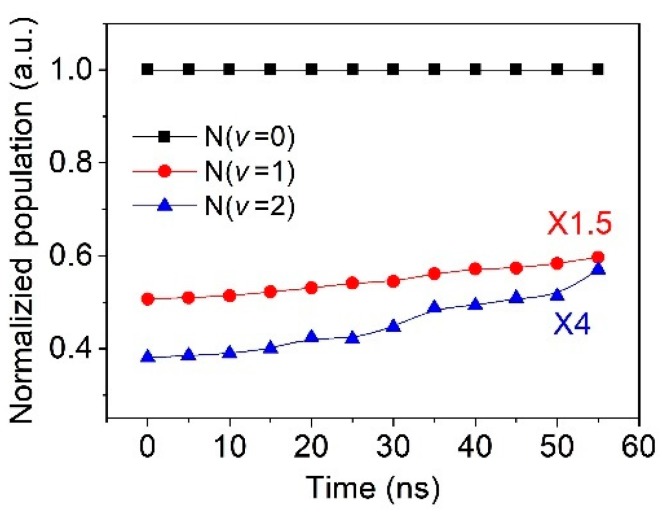
Temporal dependence of the relative vibrational populations of N_2_ (C^3^Π_u_, v = 0), N_2_ (C^3^Π_u_, v = 1), and N_2_ (C^3^Π_u_, v = 2).

Figure 7 shows the temporal distribution of the relative vibrational populations of N_2_ (C^3^Π_u_, v = 1), N_2_ (C^3^Π_u_, v = 2) with the population of N_2_ (C^3^Π_u_, v = 0) normalized to 1. The discharge was operated at the pulse peak voltage, pulse repetition rate, and electrode gap distance of 26 kV, 100 Hz, and 5 mm, respectively. The time in the Figure is counted from the initial moment of the discharge. During the discharge in each pulse, the populations of higher vibrational level N_2_ (C^3^Π_u_) exhibits an obvious increase with the discharge time. At the time of 55 ns, the population of N_2_ (C^3^Π_u_, v = 1) and N_2_ (C^3^Π_u_, v = 2) increase to 0.39 and 0.14 (at 0 ns they are 0.34 and 0.096), respectively. Obviously, the increase of the population of N_2_ (C^3^Π_u_, v = 2) between the times t = 0 and t = 55 ns is larger than that of N_2_ (C^3^Π_u_, v = 1).

During the discharge, the excited molecules N_2_ (C^3^Π_u_, v = 0, v = 1, v = 2) are generated by the electron impact excitation processes with ground state nitrogen molecules N_2_ (X^1^Σ_g_^+^).

(4) $$N_{2}(X^{1}\Sigma_{g}^{+})+e\to N_{2}(C^{3}\Pi_{u},\nu \;=\;0–3)\;k_{\nu ’}$$

The reaction rate constants $k_{\nu \prime }$ for different vibrational levels v = 0, v = 1, v = 2 can be expressed as a function of the cross sections, which is influenced by the *E/N*. It should be noted that the distribution of the vibrational levels does not reach the equilibrium after the fast electron impact reaction. The overpopulations of high vibrational levels exist for the high electron temperature in NPDBD [44].

During the energy transfer processes, there are two approaches leading the obvious increase of vibrational populations of N_2_ (C^3^Π_u_, v = 1) and N_2_ (C^3^Π_u_, v = 2), i.e., the electron impact vibrational excitation (Equation (5)) and vibrational–vibrational (V–V) energy exchange (Equations (6) and (7)). Since the vibrational–rotational (V–R) energy exchange has a time scale of the order of microsecond to millisecond, it can be neglected during the discharge [45].

*e* + N_2_ (C^3^Π_u_, v = 0) → *e* + N_2_ (C^3^Π_u_, v > 0) (5)

N_2_ (C^3^Π_u_, v = 0) + N_2_ (v > 1) → N_2_ (C^3^Π_u_, v = 1) + N_2_ (v–1) (6)

N_2_ (C^3^Π_u_, v = 1) + N_2_ (v > 2) → N_2_ (C^3^Π_u_, v = 2) + N_2_ (v–1) (7)

**Figure 8:**
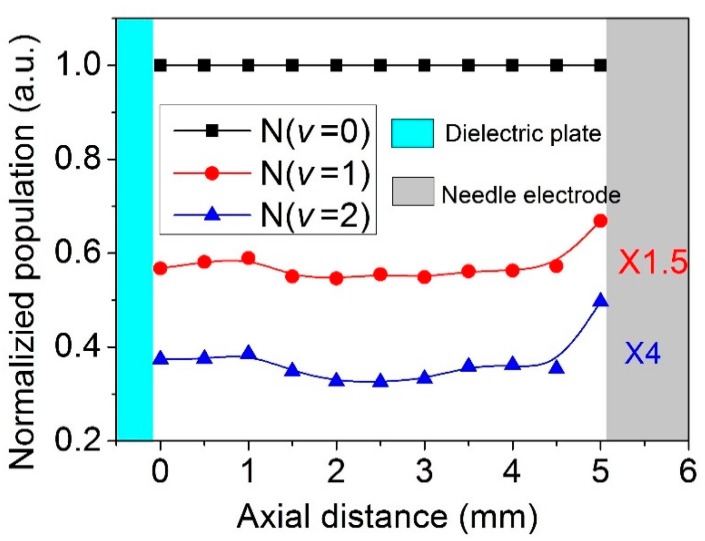
Spatial distribution of the relative vibrational populations of N_2_ (C^3^Π_u_, v = 0), N_2_ (C^3^Π_u_, v = 1), and N_2_ (C^3^Π_u_, v = 2).

Fundamentally, the energy of vibration excitation state of N_2_ (C^3^Π_u_, v) is granted from the electron energy, so the population of vibrational excited state of nitrogen molecules can be availabel for some insight into electron temperature for an integrated time [34,46]. Figure 8 shows the spatial distribution of the relative vibrational populations of N_2_ (C^3^Π_u_, v = 0), N_2_ (C^3^Π_u_, v = 1), N_2_ (C^3^Π_u_, v = 2). For a better comparison, the relative vibrational population of N_2_ (C^3^Π_u_, v = 0) is normalized to 1. The positions of needle electrode and dielectric plate are 5 mm and 0 mm, marked with different color lump in the figure. The discharge is operated at 26 kV pulse peak voltage, 100 Hz pulse repetition rate, and 5 mm electrode gap distance.

From the spatial distribution of the relative vibrational populations, it is shown that both the relative populations of N_2_ (C^3^Π_u_, v’ = 1) and N_2_ (C^3^Π_u_, v’ = 2) exhibit a maximum near the needle tip and decrease gradually with the distance from the needle electrode. However, near the plate electrode, the populations of N_2_ (C^3^Π_u_, v’ = 1) and N_2_ (C^3^Π_u_, v’ = 2) present a slight increase. Since the vibration distribution of N_2_ is mainly caused by the electron impact vibration excitation in our experiment, the populations of N_2_ (C^3^Π_u_, v’ = 1) and N_2_ (C^3^Π_u_, v’ = 2) can show the maps of *E/N*. In addition, near the needle electrode, the high *E/N* is beneficial to the breakdown of the gas gap and the slight increase of the electric field near the dielectric plate, which can excite the surface discharge on the dielectric plate.

### 3.4. Calculation of Reduced Electric Field in NPDBD

In NPDBD at atmospheric air, it is proposed that the *E/N* can be calculated using the intensity ratio of second positive system (SPS) of N_2_ to first negative system (FNS) of $N_{2}{}^{+}$ [36]. Therefore, the evolution of *E/N* can be represented by the temporal–spatial emission spectra ratio of N_2_ (C^3^Π_u_ → B^3^Π_g_) and $N_{2}{}^{+}(B^{2}\Sigma_{u}^{+}$ → X^2^Σ_g_^+^) [37]. For the detected spectra of N_2_ (C^3^Π_u_ → B^3^Π_g_, v’, v”) and $N_{2}{}^{+}(B^{2}\Sigma_{u}^{+}$ → X^2^Σ_g_^+^,0–0, 391.4 nm), the corresponding population of upper vibrational excited state N_2_ (C^3^Π_u_, v = 0–3) and $N_{2}{}^{+}(B^{2}\Sigma_{u}^{+}$, v = 0) are determined by the electron impact vibrational excitation from ground state nitrogen molecule N_2_ (X^1^Σ_g_^+^) by the Equations (4) and (8):

(8) $$e+N_{2}(X^{1}\Sigma_{g}^{+})\to N_{2}^{+}(B^{2}\Pi_{u}^{+},\nu \;=\;0)\;\;k_{i}$$

The reaction rates *k_v’_*(v*’* = 0–3) and *k*_i_ can be calculated by the cross sections from BOLSIG^+^ and database LXcat as Equation (9):

(9) $$k=\gamma \int_{0}^{\infty }\varepsilon \sigma \left(\varepsilon \right)F_{0}d\varepsilon ,$$

where $\gamma =(2{e/m)}^{1/2}$ is a constant, $\varepsilon =(v/\gamma {)}^{2}$ is the electron energy in eV, in which v is electron velocity, and the function *F*_0_ is the isotropic part of electron distribution *F*. For the cross sections σ(ε), it can be obtained by the relationship with the reduce electric field (*E/N*) in function (8):

(10) $$-\frac{\gamma e^{2}}{3}{\left(\frac{E}{N}\right)}^{2}\frac{d}{d\varepsilon }(\frac{\varepsilon }{Q}\frac{dF_{0}}{d\varepsilon })=C_{0}[F_{0},\{\sigma (\varepsilon )\}]-\frac{\Omega }{NC}\varepsilon^{1/2}F_{0},$$

where the momentum cross section Q can be defined as $Q=\sigma (\varepsilon )+\Omega /N\gamma \varepsilon^{1/2}$, the energy distribution $F_{0}$ is constant in time and space, and $C_{0}$ is the change in *F*_0_ due to collisions [47]. For an accurate calculation, the cross sections of the vibrational structure of N_2_ (C^3^Π_u_, *v* = 0–3) was calculated using the Frank Condon factors of the N_2_ (C^3^Π_u_ → B^3^Π_g_) listed in Table 1. 

For the depopulation, the N_2_ (C^3^Π_u_, v = 0–3) and $N_{2}{}^{+}(B^{2}\Sigma_{u}^{+}$, v = 0) can be quenched by the spontaneous radiative depopulation and collisions with heavy particles by Equations (11)–(16):

(11) $$N_{2}(C^{3}\Pi_{u},v\prime )\to N_{2}(B^{3}\Pi_{u},v\prime \prime )+hv_{v\prime v\prime \prime }\;k=1/\tau_{v\prime v\prime \prime }$$

(12) $$N_{2}(C^{3}\Pi_{u},v\prime =0-3)+N_{2}\to \mathrm{products}\;k_{q,N_{2}}$$

(13) $$N_{2}(C^{3}\Pi_{u},v\prime =0-3)+O_{2}\to \mathrm{products}\;k_{q,O_{2}}$$

(14) $$N_{2}{}^{+}(B^{2}\Sigma_{u}{}^{+},v\prime =0)+N_{2}\to \mathrm{products}\;k_{q,N_{2}}$$

(15) $$N_{2}{}^{+}(B^{2}\Sigma_{u}{}^{+},v\prime =0)+O_{2}\to \mathrm{products}\;k_{q,O_{2}}$$

(16) $$N_{2}{}^{+}(B^{2}\Sigma_{u}{}^{+},v\prime =0)+N_{2}+M\to N_{4}{}^{+}+M\;k_{conv}$$

The radiative lifetimes and deactivation rate constants of different separated vibrational state of N_2_ (C^3^Π_u_) and $N_{2}{}^{+}(B^{2}\Sigma_{u}^{+}$) are shown in Table 2:

Therefore, the change in the excited state particle [N_exc_] concentrations of N_2_ (C^3^Π_u_, v’ = 0,1,2) and $N_{2}{}^{+}(B^{2}\Sigma_{u}^{+}$) can be expressed as the production of the electron impact processes and the depopulation as (17a) and (17b), where the associative conversion by three-body collisions (Equation (16)) is considered in the quenching of the nitrogen ions:

(17a) $$\frac{d[N_{exc}]}{dt}=k_{exc}n_{e}[N_{2}]-\frac{1}{\tau }[N_{exc}]-k_{q,N_{2}}[N_{2}][N_{exc}]-k_{q,O_{2}}[O_{2}][N_{exc}]$$

(17b) $$\begin{array}{l} \frac{d[N_{exc}]}{dt}=k_{exc}n_{e}[N_{2}]-\frac{1}{\tau }[N_{exc}]-k_{q,N_{2}}[N_{2}][N_{exc}]-k_{q,O_{2}}[O_{2}][N_{exc}]\\ -k_{conv}[N_{2}][M][N_{exc}] \end{array}$$

For the selected vibrational transition of SPS of N_2_ and FNS of $N_{2}{}^{+}$, the emission intensity expresses as:

(18) $$I_{v\prime v\prime \prime }=h\nu_{v\prime v\prime \prime }N_{v\prime v\prime \prime }\tau_{v\prime v\prime \prime }A_{v\prime v\prime \prime }=h\nu_{v\prime v\prime \prime }n_{e}[N_{2}]gA_{v\prime v\prime \prime },$$

where the probability of *A*_v’v”_ is calculated by $q_{v\prime v\prime \prime }$ using function $A_{v\prime v\prime \prime }=\frac{q_{v\prime v\prime \prime }\nu_{v\prime v\prime \prime }^{3}}{\Sigma_{v\prime \prime }q_{v\prime v\prime \prime }\nu_{v\prime v\prime \prime }^{3}}$, the corrected g-function can be expressed by $g={\left(1-\tau (k_{q,N_{2}}[N_{2}]+k_{q,O_{2}}[O_{2}])[M]\{+k_{conv}[N_{2}]{\left[M\right]}^{2}\}\right)}^{-1}$, and the number density of [N_2_] and [O_2_] are estimated to be equal to 2.12 × 10^19^ cm^−3^ and 5.6 × 10^18^ cm^−3^ at atmospheric air.

Thus, the intensity ratio *R_391_/R*_v’v”_ is expressed as:

(19) $$R_{391}/R_{v\prime v\prime \prime }=I_{391}/I_{v\prime v\prime \prime }={\left(\frac{\lambda_{391}}{\lambda_{v\prime v\prime \prime }}\right)}^{-1}\frac{A_{391}}{A_{v\prime v\prime \prime }}\frac{g_{391}}{g_{v\prime v\prime \prime }}\frac{k_{391}}{k_{v\prime v\prime \prime }}$$

**Figure 9:**
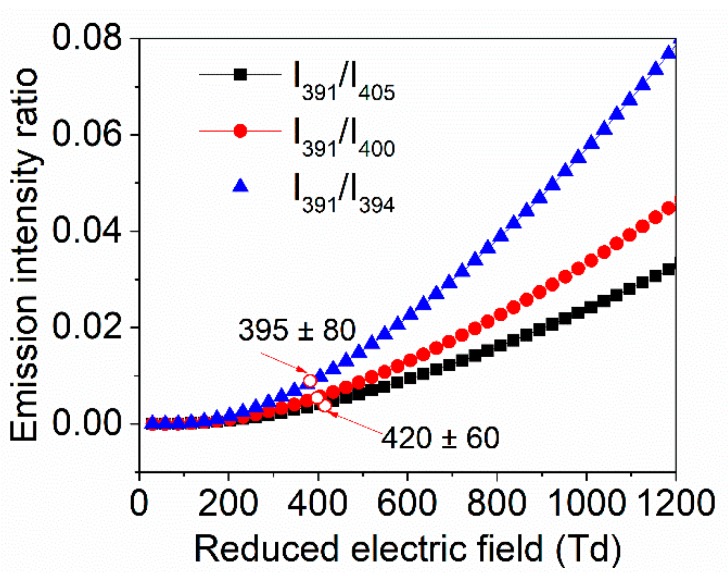
The intensity ratios of *I*_391_/*I*_405_*, I*_391_/*I*_400_*,* and *I*_391_/*I*_394_ as function of *E/N.*

As described in Equations (8) and (9), the excitation rate constants of electron impact processes depend on the *E*/*N* only, that means, the intensity ratios of *I*_391_/*I*_405_*, I*_391_/*I*_400_*,* and *I*_391_/*I*_394_*,* etc., can be used to calculate the reduce electric field. The measured intensity ratios for these vibrational bands at a position of 5 mm from the needle electron tips and at the time of 15 ns are marked in Figure 9, which are about 420 Td, 400 Td, and 395 Td, respectively.

### 3.5. Temporal Evolution of Reduced Electric Field E/N

**Figure 10:**
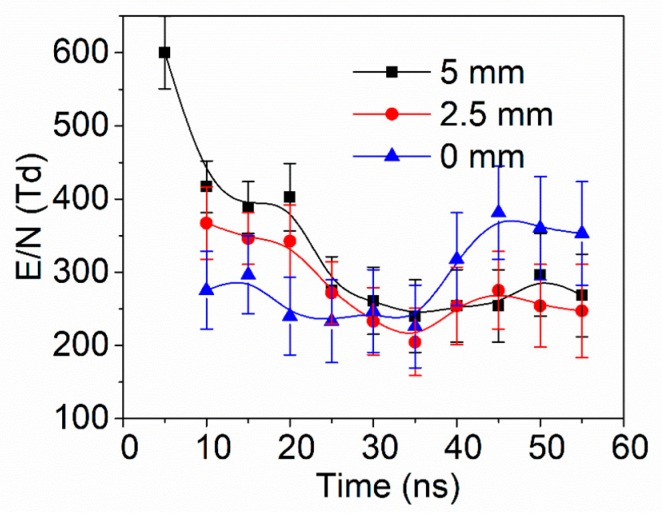
The temporal evolution of *E/N* at 0 mm, 2.5 mm, 5 mm from the plate electrode in NPDBD.

Figure 10 shows the temporal evolution of *E/N* at various positions (0 mm, 2.5 mm, 5 mm from the plate electrode) in NPDBD. In the measurement, the pulse peak voltage, pulse repetition rate, and electrode gap are also kept at 26 kV, 100 Hz, and 5 mm, respectively. It shows that the curves of *E/N* present different tendencies compared with the waveform of pulse voltage. In the region near the needle tip (5 mm from plate electrode), the *E/N* presents a maximum at 5 ns, which is about 590 ± 80 Td. When the initial streamer channel was formed, the *E/N* decreases sharply in the discharge duration accompanied with the sharply increase of plasma optical emission intensity. In the period of 15–35 ns, the *E/N* is about 270–420 Td, which is only about 1/2–2/3 compared with the *E/N* at 5 ns. In the study of Fridman [48], the *E/N* of diffuse regime in NPDBD is only about 1/2 of the streamer regime. Therefore, the low *E/N* at t = 15 ns indicates that mode transition from streamer regime to diffuse regime is accomplished. At the central position of the electrode gap (2.5 mm from plate electrode), the *E/N* is much weaker compared with that near the needle tip. The distorted high *E/N* cannot be observed, instead, the maximum *E/N* is about 370 Td at the time of 10 ns, then the *E/N* decreases gradually with the transition from streamer to diffuse discharge. At the time of 25 ns, the *E/N* at the central position is almost kept consistent with the *E/N* at the position near the needle tip. Near the surface of dielectric plate (0 mm), the *E/N* presents a completely different evolution tendency. For the discharge duration of 5–25 ns, it decreases gradually and keeps a low value (220–350 Td). However, once the diffuse discharge extinguished and the surface discharge begins to propagate to the outside direction (t = 30–50 ns), the *E/N* increases with the duration of discharge time obviously. At the time of 50 ns, the *E/N* on the surface of dielectric plate is about 365 Td, which is about 100 Td higher than the *E/N* at the positions of needle tip and 50 Td higher than that at central place of electrode gap.

In the NPDBD, the *E/N* is determined by the overlap of applied pulsed electric field and the built-in electric field formed by the space charge in the plasma region and memory charge on the surface of dielectric plate. At the initial time, a distorted high *E/N* is formed near the needle electrode due to the extremely asymmetrical electrode configuration, which excites the initial streamer from needle electrode to plate electrode. Caused by the high conductivity in the streamer channel, the *E/N* decreases sharply when the gas gap is broken down. Once the diffuse discharge forms, the *E/N* in the whole electrode gap is rebuilt, it almost equally distributes in the axial distance. The memory charges on the surface of dielectric plate can be erased by the plasma, which can build an electric field along dielectric plate. This horizontal electric field drives the surface barrier discharge propagating in the radial direction after the volume discharge extinguished. 

## 4. Conclusions

Evolution dynamic process in a discharge pulse is observed by one-shot ICCD images. Three main stages in NPDBD are distinguished, which are the streamer breakdown from needle tip to plate electrode, the regime transition from streamer to diffuse, and the propagation of surface discharge on the plate electrode surface. At the beginning of the discharge, the *E/N* near the needle tip can be estimated to about 590 Td. This high *E/N* excites the initial breakdown as a positive streamer regime. The streamer builds up a new electric field with radial direction and pre-photoionizes the surrounding air to drive the subsequent breakdown. Hence, an abundance of fine secondary streamer channels around the initial streamer form at t = 10 ns, contributing to the transition to diffuse regime. By measuring the temporal–spatial resolved spectra, it is found that the spectra of $N_{2}{}^{+}(B^{2}\Sigma_{u}^{+}$ → X^2^Σ_g_^+^) and N_2_ (C^3^Π_u_ → B^3^Π_g_) present obvious different evolution tendencies. The band of $N_{2}{}^{+}(B^{2}\Sigma_{u}^{+}$ → X^2^Σ_g_^+^), indicator of high *E/N*, is mainly emitted from the region near the needle tip in the initial period of the breakdown process. The energy distribution, the relative vibration population of N_2_ (C^3^Π_u_, *v* = 0,1,2), and the *E/N* are calculated. The populations of N_2_ (C^3^Π_u_, v = 1,2) increase with the discharge duration time. The increase of N_2_ (C^3^Π_u_, v = 2) is larger than that of N_2_ (C^3^Π_u_, v = 1). The evolutions of *E/N* are calculated using the temporal–spatial resolved spectra of $N_{2}{}^{+}(B^{2}\Sigma_{u}^{+}$ → X^2^Σ_g_^+^) and N_2_ (C^3^Π_u_ → B^3^Π_g_). It is found that *E/N* near the plate electrode (0 mm), at the middle of the electrode gap (2.5 mm), near the needle electrode (5 mm), present different tendencies with the waveform of pulse voltage. A distorted high *E/N* can be observed near the needle electrode at 5 ns. At the time of 10−25 ns, the *E/N* decreases to about 270–320 Td, which indicates that mode transition from streamer regime to diffuse regime is accomplished. Near the surface of dielectric plate, the *E/N* decreases gradually and keeps a low value at the time of 10–25 ns, but increases obviously after the diffuse discharge extinguished (at the time of about 30–50 ns). At the time of 50 ns, the *E/N* on the surface of dielectric plate is about 100 Td higher than the *E/N* at the positions of needle tip and 50 Td higher than that at central place of electrode gap. It drives the surface barrier discharge propagating in the radial direction along the dielectric plate. 

## Figures and Tables

**Table 1:** Franck-Condon factors for the C^3^Π_u_-B^3^Π_g_ (Second Positive System) [45].

v”	v’ = 0	v’ = 1	v’ = 2	v’ = 4	v’ = 5
0	4.55 × 10^−1^	3.88 × 10^−1^	1.34 × 10^−1^	2.16 × 10^−2^	1.16 × 10^−3^
1	3.31 × 10^−1^	2.92 × 10^−2^	3.35 × 10^−1^	2.52 × 10^−1^	5.66 × 10^−2^
2	1.45 × 10^−1^	2.12 × 10^−1^	2.30 × 10^−2^	2.04 × 10^−1^	3.26 × 10^−1^
3	4.94 × 10^−2^	2.02 × 10^−1^	6.91 × 10^−2^	8.81 × 10^−2^	1.13 × 10^−1^
4	1.45 × 10^−2^	1.09 × 10^−1^	1.69 × 10^−1^	6.56 × 10^−3^	1.16 × 10^−1^
5	3.87 × 10^−3^	4.43 × 10^−2^	1.41 × 10^−1^	1.02 × 10^−1^	2.45 × 10^−3^
6	9.68 × 10^−4^	1.52 × 10^−2^	7.72 × 10^−2^	1.37 × 10^−1^	4.70 × 10^−2^

**Table 2:** Radiative and deactivation parameters of N_2_ (C^3^Π_u_) and ${N_{2}}^{+}(B^{2}\Sigma_{u}^{+})$.

	N_2_ (C^3^Π_u_, v = 0)	N_2_ (C^3^Π_u_, v = 1)	N_2_ (C^3^Π_u_, v = 2)	N_2_ (C^3^Π_u_, v = 3)	${N_{2}}^{+}(B^{2}\Sigma_{u}^{+},\;v\;=\;0)$
τ (ns) [37]	42	41	39	41	62
*A*_v’v”_	0.05 (0–3)	0.11 (1–4)	0.14 (2–5)	0.14 (3–6)	0.72 (0–0)
*k*_q,N2_ (10^−10^ cm^3^·s^−1^)	0.13	0.29	0.46	0.43	2.1
*k*_q,N2_ (10^−10^ cm^3^·s^−1^)	3.0	3.1	3.7	4.3	5.1
*k_conv_,* (10^−29^ cm^3^·s^−1^)	-	-	-	-	5.0
*g*	0.012	1.0 × 10^−2^	8.3 × 10^−3^	7.3 × 10^−3^	2.2 × 10^−3^
